# Prevalence of Electrographic Seizures in Hospitalized Patients With Altered Mental Status With No Significant Seizure Risk Factors Who Underwent Continuous EEG Monitoring: A Retrospective Study

**DOI:** 10.7759/cureus.55903

**Published:** 2024-03-10

**Authors:** Elena Garrido, Amir Adeli, Marco Echeverria-Villalobos, Juan Fiorda, Yousef Hannawi

**Affiliations:** 1 Department of Anesthesiology, The University of Iowa Carver College of Medicine, Iowa City, USA; 2 Department of Neurology, Division of Epilepsy, The Ohio State University Wexner Medical Center, Columbus, USA; 3 Department of Anesthesiology, The Ohio State University Wexner Medical Center, Columbus, USA; 4 Department of Neurology, Division of Cerebrovascular Diseases and Neurocritical Care, The Ohio State University Wexner Medical Center, Columbus, USA

**Keywords:** altered mental status, outcome, non-convulsive seizures, continuous video eeg monitoring, electrographic seizures

## Abstract

Objectives: The objective of this study is to evaluate the prevalence of electrographic seizures in hospitalized patients with altered mental status and no significant risk factors for seizures.

Methods: We retrospectively reviewed over a six-year period (2013-2019) the medical records of all adults admitted at Ohio State University Wexner Medical Center (OSUWMC), who underwent continuous electroencephalography (cEEG) monitoring for > 48 hours. Our primary objective was to identify the prevalence of electrographic seizures in patients with altered mental status and no significant acute or remote risk factors for seizures.

Results: A total of 1966 patients were screened for the study, 1892 were excluded (96.2%) and 74 patients met inclusion criteria. Electrographic seizures were identified in seven of 74 patients (9.45%). We found a significant correlation between electrographic seizures and a history of hepatic cirrhosis, n= 4 (57%), (p=0.035), acute chronic hepatic failure during admission, 71% (n=5), (p=0.027), and hyperammonemia (p =0.009).

Conclusion: In this retrospective study of patients with altered mental status and no significant acute or remote risk factors for seizures who underwent cEEG monitoring for > 48 hours, electrographic seizures were identified in 9.45%. Electrographic seizures were associated with hepatic dysfunction and hyperammonemia. Based on our results, cEEG monitoring should be considered in patients with altered mental status and hepatic dysfunction even in the absence of other seizure risk factors.

## Introduction

Continuous electroencephalography (cEEG) is an invaluable tool to detect seizures in patients who are critically ill and have altered mental status [1], as clinical signs (like ictal nystagmus, facial twitching) are often minimal or may not be present [2]. In contrast, some of these patients exhibit abnormal movements that are not seizure-related but could be interpreted as such [3]. Without the use of cEEG, these patients may be unnecessarily treated with anti-seizure medications [4]. Notably, patients with electrographic seizures and electrographic status-epilepticus stay longer in the intensive care unit (ICU) and have a greater incidence of hospital mortality, highlighting the importance of early detection and treatment of these seizures [5]. Thus, cEEG provides real-time information on brain function, which is critical to detect electrographic seizures as well as to differentiate abnormal non-epileptic movements from epileptic ones [2].

The role of cEEG in identifying seizures has been well established in patients with acute ischemic stroke [6-9], intraparenchymal hemorrhage [8,10-13], aneurysmal subarachnoid hemorrhage [7,14,15], brain tumors [6,9], moderate to severe traumatic brain injury (TBI) [7], central nervous system infections and inflammation [16], and anoxic brain injury [1]. Moreover, the use of cEEG to evaluate encephalopathy in critically ill patients without apparent brain injury is less well established [17], although evidence supports its use in such cases. Studies in patients with sepsis, chronic renal failure, or critically ill patients with altered mental status have highlighted a significant risk for developing nonconvulsive seizures due to the development of acute encephalopathy. For example, uremic encephalopathy is a cerebral dysfunction seen in the setting of acute or chronic renal failure and is usually associated with a very low glomerular function rate. This is likely caused by uremic toxins leading to neurotoxic effects through excitatory N-methyl-d-aspartate (NMDA) receptors and inhibition of γ-aminobutyric acid (GABA) receptors. In the case of patients with sepsis-associated encephalopathy, the exact pathophysiologic mechanisms for developing nonconvulsive seizures have not been elucidated, and several hypotheses remain to be clarified [18,19]. In addition, a retrospective study found that chronic hepatic disease tended to be associated with electrographic seizures (ESz) in surgical ICU patients with altered mental status and without primary acute brain injury [8]. However, it is unclear what symptoms may be indicative of seizure risk and support the use of cEEG to monitor patients.

In the last several years, there has been growing recognition of cephalosporin-related neurotoxicity, most commonly cefepime, as a risk factor for ESz, especially in patients with renal failure, suggesting that the use of cEEG may be warranted in these patients [20,21]. The relationship between liver disease and seizures is more complex. Patients with hepatic encephalopathy may present epileptiform abnormalities and seizures with unknown pathophysiology [22-24]. In contrast, a more recent retrospective cohort study in a large sample of elderly patients suggested that the presence of liver disease was not associated with seizures. In this study, only patients with cirrhosis more frequently exhibited status epilepticus, although these patients also exhibited other risk factors for seizures (e.g., TBI, CNS infections) [24]. In clinical practice, neurologists are commonly consulted on hospitalized patients with altered mental status and must determine whether continuous EEG monitoring would be an appropriate test for diagnostic evaluation. The EEG Taskforce of the American Clinical Neurophysiology Society has established recommendations on the use of video cEEG in critically ill adults with well-defined indications. This includes recommending use in patients with fluctuating mental status, agitation, lethargy, fixed or fluctuating neurologic deficits, obtundation, and coma [25]. To identify at least 88% to 93% of first seizures, it is recommended that EEG recordings are obtained for 24 to 48 hours [12]. However, given the limited availability and labor-intensive nature of cEEG, it is of paramount importance to determine the cohort of patients most in need of cEEG monitoring due to associated complications that put them at high risk of seizures [12,26].

We hypothesized that the prevalence of ESz would be very low in patients with an altered mental status with no significant acute or remote risk factors for seizures and that continuous EEG monitoring would not provide data that would warrant a change in clinical management. We therefore conducted a retrospective chart review to identify the prevalence of electrographic seizures in patients with altered mental status and no known acute or remote risk factors for seizures.

This article was previously posted to the Research Square preprint server on October 13, 2023.

## Materials and methods

This is an observational retrospective chart review of a cohort of adult patients > 18 years of age who underwent continuous video cEEG for > 48 hours between January 1st, 2013 and December 31st, 2019 at Ohio State University Wexner Medical Center (OSUWMC). We obtained data from the electronic health records from The Ohio State University Wexner Medical Center, one of the largest healthcare providers in Ohio. The Institutional Review Board (IRB) approved the study with number 2020H0040. The requirement for informed consent was waived because the risk for patients was minimal.

We identified electrographic seizures within our Comprehensive Epilepsy Center electronic database which included start time, end time, and date documentation of all in-patient electroencephalogram monitoring. We obtained the medical information of each patient by reviewing the electronic medical records (Epic; Verona, WI, USA). Patients were included if they underwent long-term cEEG monitoring for evaluation of altered mental status, which consisted of patient lethargic, stuporous, or comatose (International Classification of Diseases, Tenth Revision, Clinical Modification [ICD-10-CM] diagnosis of R41.82), either as the primary cause of admission or occurring through the course of their hospitalization. Patients with the following seizure risk factors were excluded: acute or remote history of ischemic stroke, hemorrhagic stroke/intracerebral hemorrhage, subarachnoid hemorrhage, epidural hematoma, subdural hematoma, severe traumatic brain injury, central nervous system infection, neurosurgical (brain) procedure, cardiac/respiratory arrest, acute or chronic kidney injury and concomitant administration of cephalosporin class of antibiotics, alcohol, or drug dependence, and established epilepsy.

Regarding patient selection criteria and data extraction, two reviewers (E.G., A.A.) independently performed the patient study selection and data extraction using individual clinical criteria. The obtained data were subsequently converted into a Microsoft Excel spreadsheet for further processing of the data. Extracted information included baseline patient characteristics, and data such as age, gender, and medical history (including diabetes, chronic renal failure, and chronic hepatic failure). Clinical data at the initiation of cEEG included suspected in-hospital clinical seizures, coma, circulatory shock requiring vasopressors, pneumonia, and the need for mechanical ventilation.

cEEG was recorded using 21 standard scalp electrodes according to the International 10-20 System, placed by board-certified EEG technologists. cEEG was interpreted by board-certified epileptologists and reviewed by one of the main authors (A.A.) who determined whether inclusion criteria were met. ESz were defined based on Salzburg criteria [27]. Clinical seizures were defined as clinically observable seizure activity occurring in conjunction with epileptiform abnormalities on cEEG. Data was obtained from consultants and primary team notes, cEEG reports, and discharge summaries. Laboratory results, imaging reports, and inpatient medication lists were also reviewed. The discharge status of the patient was simply classified as good (either home or rehabilitation) or poor (death during hospital stay).

Statistical analysis

Summary statistics were calculated for the overall study sample and stratified by seizure status. Categorical measures are presented as counts and percentages. Due to non-normal distributions, all continuous variables are presented as medians and interquartile ranges. P-values were generated to compare patients who did and did not experience seizures using Wilcoxon rank sum tests for continuous measures and Fisher’s Exact Test for categorical measures. A significance threshold of 0.05 two-sided was used for all statistical tests. All statistical analyses were done using R Statistical Software (version 4.1.3; R Foundation for Statistical Computing, Vienna, Austria) and gtsummary software package.

## Results

Patient characteristics

**Figure 1:**
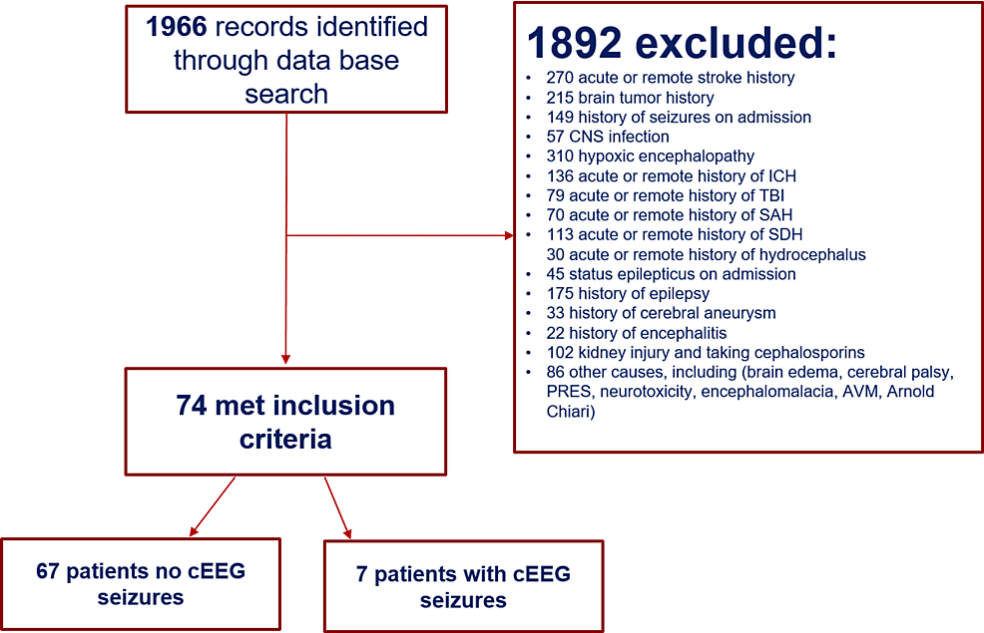
Flow diagram detailing eligible patients in the study cEEG: Continuous electroencephalography; CNS: central nervous system; PRES: posterior reversible encephalopathy syndrome; ICH: Intracerebral hemorrhage; TBI: traumatic brain injury; SAH: subarachnoid hemorrhage; SDH: subdural hematoma; AVM: arteriovenous malformation

We identified through database search a total of 1966 patients with altered mental status who underwent cEEG monitoring for > 48 hours from January 2013 to December 2019. Of these 1966 patients, 1892 (96.2%) did not meet the inclusion criteria at the time cEEG was ordered (Figure 1). Seventy-four patients (3.7%) who fulfilled the inclusion criteria were identified. Patient characteristics are presented in Table 1.

**Table 1:** Patients’ baseline characteristics ^1^Median (IQR); no. (%); ^2^Wilcoxon rank sum test; Fisher's exact test; ^3^Including NASH cirrhosis, TIPS; ^4^Including chemotherapy ^5^including STEMI, heart failure; ^6^Including aspiration pneumonia, acute respiratory distress syndrome, and chronic pulmonary disease exacerbation; ^7^Including esophageal fistula, cardiac transplant, lung transplant, exploratory laparotomy, and incarcerated hernia; ^8^Including urinary tract infection and septic shock NASH: Nonalcoholic steatohepatitis; STEMI: ST-elevation myocardial infarction

Characteristic^1^	Overall (n=74)	Without electrographic seizures (n=67)	With electrographic seizures (n=7)	p-value^2^
Age, Years (IQR)	62 (54-70)	63 (53-70)	61 (60-66)	0.6
Female Gender	36 (49%)	33 (49%)	3 (43%)	>0.9
Hypertension	40 (54%)	37 (55%)	3 (43%)	0.7
Diabetes Mellitus	31 (42%)	28 (42%)	3 (43%)	>0.9
Atrial Fibrillation	17 (23%)	15 (22%)	2 (29%)	0.7
Coronary Artery Disease	14 (19%)	13 (19%)	1 (14%)	>0.9
Chronic Renal Failure	15 (20%)	14 (21%)	1 (14%)	>0.9
Hepatic Cirrhosis	16 (22%)	12 (18%)	4 (57%)	0.035
Oncologic	19 (26%)	17 (25%)	2 (29%)	>0.9
End Stage Renal disease (ESRD)	11 (15%)	11 (16%)	0 (0%)	0.6
Primary Admission Diagnosis				0.2
Acute Hepatic Failure^3^	3 (4.1%)	2 (3.0%)	1 (14%)	
Acute Renal Failure	1 (1.4%)	1 (1.5%)	0 (0%)	
Altered Mental Status	27 (36%)	24 (36%)	3 (43%)	
Cardiology	2 (2.7%)	2 (3.0%)	0 (0%)	
Oncologic ^4^	7 (9.5%)	5 (7.5%)	2 (29%)	
Others ^5^	6 (8.1%)	5 (7.5%)	1 (14%)	
Respiratory Failure ^6^	8 (11%)	8 (12%)	0 (0%)	
Sepsis	16 (22%)	16 (24%)	0 (0%)	
Surgical^7^	4 (5.4%)	4 (6.0%)	0 (0%)	
Suspected clinical seizures prior cEEG	16 (22%)	13 (19%)	3 (43%)	0.2
Medical Complications during admission				
Acute Kidney Injury	44 (58%)	39 (58%)	5 (71%)	0.7
Sepsis ^8^	34 (46%)	31 (46%)	3 (43%)	>0.9
Ventilator Associated Pneumonia	29 (39%)	26 (39%)	3 (43%)	>0.9
Acute Hepatic Failure	23 (31%)	18 (27%)	5 (71%)	0.027
Mechanical Ventilation	49 (66%)	43 (64%)	6 (86%)	0.4
Received Any Type of Dialysis	22 (30%)	20 (30%)	2 (29%)	>0.9
Vasopressors Use	26 (35%)	22 (33%)	4 (57%)	0.2
Death at Hospital Discharge	22 (30%)	18 (27%)	4 (57%)	0.2
Death at Hospital Discharge OR Death at Three Months	27 (36%)	22 (33%)	5 (71%)	0.09

Of these seventy-four patients, seven patients were found to have electrographic seizures (9.45%). The median age for patients with electrographic seizures was 61 (60-66) years of age and without electrographic seizures was 63 (53-70) (p=0.6). Of patients with electrographic seizures, there was a significant association with history of chronic liver disease, predominantly cirrhosis, acute hepatic failure during admission, and hyperammonemia compared with patients without electrographic seizures (p=0.035; p=0.027; p=0.009, respectively) (Tables 1, 2).

**Table 2:** Laboratory results in the total cohort of patients ^1^Median (IQR); ^2^Wilcoxon rank sum test; Fisher's exact test; ^3^Upper limit of normal 60 µmol/L

Blood test parameters	Total cohort (n=74)	Without seizures (n=67)	With seizures (n=7)	p-value ^2^
Venous Lactate^1 ^─ mmol/L	2.00 (1.40-4.29)	1.99 (1.35-4.06)	2.54 (1.82-6.18)	0.3
Missing Value ─ no.	16	16	0	
Creatinine^1^ ─ mg/dL	1.22 (0.78-2.18)	1.27 (0.78-2.22)	0.86 (0.79-1.30)	0.3
Missing Value ─ no.	1	1	0	
Ammonia^1,3^ ─ µmol/L	48 (34-81)	42 (34-63)	252 (129-291)	0.009
Missing value ─ no.	21	20	1	

No differences were seen in other medical complications, such as acute kidney injury, sepsis, need for ventilation or need for vasopressors in patients with or without electrographic seizures (Table 1). There were no differences between the two groups with regards to death at discharge or at three months (p=0.09) (Table 1).

All seven patients with electrographic seizures underwent head CT. In one patient, head CT identified diffuse brain edema and in the remaining six patients, it was normal. Six patients underwent brain MRI following completion of cEEG, which showed hyperammonemia-related changes in two patients (p=0.2) (Table 3 and Table 4). At the time cEEG was placed, electrographic seizures were detected in three patients during the first six hours, one additional one from 6 to 12 hours, two additional patients from 12-24 h, and one additional patient from 24-48 hours (Table 4).

**Table 3:** Brain image results ^1^no. (%); ^2^Fisher's exact test

Neuroimaging tests	Total cohort (n=74)	Without seizures (n=67)	With seizures (n=7)	p-value ^2^
CT Scan Results─ no. (%)				0.4
Normal^1^	69 (93%)	63 (94%)	6 (86%)	
Abnormal^1^	5 (6.8%)	4 (6.1 %)	1 (14%)	
MRI Results─ no. (%)				0.2
Normal^1^	33 (45%)	30 (45%)	3 (43%)	
Abnormal^1^	14 (19%)	11 (16%)	3 (43%)	
No MRI^1^	27 (36%)	26 (39%)	1 (14%)	

**Table 4:** Characteristics of patients with electrographic seizures cEEG: Continuous electroencephalography

Patient #	Primary Cause of Admission	Reason for ordering cEEG	Lumbar Punction	Acute Hepatic Failure	Radiologic Findings	cEEG before MRI	EEG Abnormalities (Ictal)	EEG Abnormalities (Inter-Ictal)	Final Neurology Diagnosis
Patient 1	Gastrointestinal Bleed	Facial Twitching	No	Yes	Head CT: Diffuse brain edema	-	Subclinical seizures of left parietal-occipital and right hemispheric onset	Bi-hemispheric periodic epileptiform discharges	Hepatic Encephalopathy
Patient 2	Altered Mental Status with Sepsis	Facial Twitching	No	Yes	MRI: Hyperintensity, hyperammonemia and inflammatory changes	Yes	Subclinical seizures of multi-focal onset	None	Hepatic encephalopathy
Patient 3	Altered Mental Status	New Onset Seizures	No	Yes	MRI: Hyperintensity, hyperammonemia and inflammatory changes	Yes	Subclinical seizures of left hemispheric onset	None	Hepatic encephalopathy
Patient 4	Altered Mental Status	Altered Mental Status	Yes	No	Unremarkable	Yes	3 hertz GPEDS w/triphasic morphology	Bilateral independent sharp and slow waves	Possible autoimmune encephalitis
Patient 5	Nash Cirrhosis	Seizure-Like Movement and Altered Mental Status	Yes	Yes	Unremarkable	Yes	Subclinical seizures of right temporal onset	None	Hyperammonemia encephalopathy
Patient 6	Altered Mental Status	Altered Mental Status	No	Yes	Unremarkable	Yes	Continuous diffuse spike-wave discharges	Multifocal sharp waves	Hepatic encephalopathy
Patient 7	Chemotherapy CART	Altered Mental Status	Yes	No	Unremarkable	Yes	Generalized rhythmic sharp wave activity	Intermittent GPDS w/ triphasic morphology	CART-neurotoxicity

Final neurologic diagnosis was hepatic encephalopathy in five patients, CART-neurotoxicity in one patient and in one patient no clear etiology was identified, autoimmune encephalitis was a consideration (Table 4). Specific ictal and inter-ictal EEG abnormalities for each individual patient are shown in Table 4.

## Discussion

In clinical practice, neurologists commonly encounter patients with an altered mental status and must determine whether the use of cEEG would identify findings that would lead to diagnosis and affect the clinical management of symptoms. Guidelines help physicians determine which patients may be at an elevated risk for ESz, but these emphasize known risk factors. In contrast, there is a lack of guidance for patients who exhibit no known risk factors for seizures. Therefore, we performed a retrospective chart review to evaluate the prevalence of ESz in hospitalized patients with altered mental status and no significant risk factors for seizures.

We found that most patients (96.2%) who underwent cEEG monitoring at Ohio State University Wexner Medical Center (OSUWMC) had at least one acute or remote risk factor for seizures; cEEG was rarely used in patients with an altered mental status who had no significant risk factors for seizure (used 3.7% of the time in this population), presumably due to the low pre-test probability of identifying electrographic seizures in this cohort.

Of patients with altered mental status and no significant seizure risk factors, 9.45% had ESz. The prevalence of ESz in our cohort of patients was similar to other studies; however, those studies did not consider many other seizure risk factors that could have contributed to their results [8,17,18]. In a cohort of medical ICU patients without acute neurologic injury, electrographic seizures occurred in 10% of patients [18]. Similarly, in a retrospective cohort study of surgical ICU patients without acute brain injury, ESz were detected by cEEG in 11% of patients [17]. In another study, ESz occurred in 16% of patients who were admitted to the surgical ICU and underwent cEEG due to unexplained altered mental status [8]. However, each of these studies included patients with remote risk factors for seizures, including a history of seizures [8,17,18], stroke [8,18], remote brain injury [17], and no clear indication that the patients were on cephalosporins at the time of cEEG recording [8,17,18].

In contrast to these prior reports, our study is unique because we determined the prevalence of ESz in hospitalized patients without many acute or remote risk factors for seizures, including those at risk for cephalosporin-related neurotoxicity.

We found an association between patients who had ESz and a history of chronic liver disease, acute hepatic failure during admission, or elevated ammonia levels at the time of cEEG recording. Patients with liver disease may present an increase in ammonia and there is also an increase in pro-inflammatory cytokines. These elevated levels are reported to impair postsynaptic inhibition in the cerebral cortex, and as well, to inhibit excitatory neurotransmission by a direct postsynaptic action which may lead to increased epileptogenesis. This is consistent with prior reports that found up to a third of patients presenting with acute liver failure had seizures that were associated with elevated ammonia levels [28,29]. In addition, there are case reports describing convulsive status epilepticus, epilepsia partialis continua, and electrographic seizures among patients with liver disease [22,23], and liver cirrhosis was found to reduce the seizure threshold in patients with other seizure risk factors (e.g. TBI and CNS infection), creating a favorable environment for status epilepticus [24]. In patients with chronic hepatic failure in the surgical ICU, ESz were observed more commonly [8]. However, this was in contrast with other studies that included surgical and medical ICU patients and found that chronic hepatic failure was not associated with ESz [17-18].

Similar to other studies, ESz were detected in 14% of patients in our cohort within the first hour of cEEG monitoring, in 85.7% of patients during the first 24 hours, and in 100% of patients during the first 48 hours [12,30]. It remains unclear whether ESz in critically ill patients are markers of severe illness, or if they cause secondary brain injury [17,18].

Our study has several important limitations. The first is that it was a retrospective cohort study that did not consider other confounding factors, such as selection bias, since only patients who had cEEG ordered by their clinicians were included. Second, the sample size was relatively small; a larger sample population may have demonstrated more robust findings. Third, initial diagnoses of patients upon admission were very heterogeneous and ranged from acute hepatic failure, acute respiratory failure, gastrointestinal bleeding, sepsis, or surgical and oncologic causes. Fourth, we lacked the power to perform a detailed multivariable analysis of electrographic predictors of seizures. Consequently, our data do not permit firm conclusions to be drawn regarding the clinical predictors of electrographic seizures in this cohort of patients. Fifth, lumbar puncture was not consistently performed in our cohort; therefore, some patients may have had undiagnosed CNS infections or inflammatory conditions that could have contributed to their altered mental status or seizures. Sixth, we may have more precisely identified the prevalence of electrographic seizures in patients with liver disease/hepatic encephalopathy had been excluded.

## Conclusions

Implementing cEEG as a standard monitoring method in clinical practice may be controversial. Even when it is clinically considered, it is important to identify which patients may benefit from this procedure based on how potential findings would change the clinical care these patients are otherwise receiving. Previous studies have suggested an association between hyperammonemia and subclinical seizures, which is supported by the findings in our study. We also identified both hepatic dysfunction and hyperammonemia as potential risk factors for ESz, while also excluding other confounding risk factors. Therefore, we suggest that cEEG monitoring must be considered in patients with altered mental status and concomitant hepatic dysfunction, even in the absence of other risk factors for seizure. Further prospective studies and larger sample sizes may better determine the exact prevalence of electrographic seizures in patients with no apparent seizure risk factors and subsequently provide valuable information on which cohort of patients would most benefit from cEEG monitoring.
